# Editorial: Imaging of the blood-brain barrier in Alzheimer's disease and related disorders

**DOI:** 10.3389/fnagi.2023.1252581

**Published:** 2023-07-14

**Authors:** Yuto Uchida, Xingfeng Shao, Axel Montagne, Noriyuki Matsukawa

**Affiliations:** ^1^The Russell H. Morgan Department of Radiology and Radiological Science, Johns Hopkins University School of Medicine, Baltimore, MD, United States; ^2^Department of Neurology, Nagoya City University Graduate School of Medical Sciences, Nagoya, Japan; ^3^Laboratory of FMRI Technology, Mark and Mary Stevens Neuroimaging and Informatics Institute, Keck School of Medicine, University of Southern California, Los Angeles, CA, United States; ^4^UK Dementia Research Institute, Edinburgh Medical School, University of Edinburgh, Edinburgh, United Kingdom; ^5^Centre for Clinical Brain Sciences, University of Edinburgh, Edinburgh, United Kingdom

**Keywords:** Alzheimer's disease, biomarker, blood-brain barrier, imaging, MRI, neurovascular unit

The blood-brain barrier (BBB) is a crucial physiological structure that plays a vital role in maintaining the integrity and homeostasis of the brain. It acts as a highly selective semipermeable border, formed by capillary endothelial cells, tight junctions, astrocytic end-feet, and pericytes embedded in the basement membrane, to regulate the passage of substances between the blood and the brain. The development of visualization systems using advanced imaging techniques offers new opportunities to study BBB dynamics and its contribution to neurological disorders including Alzheimer's disease and related dementias. These advancements have the potential to improve our understanding of the disease mechanisms, aid in early diagnosis, and guide the development of novel therapeutic strategies.

The purpose of this Research Topic was to encourage the proposal of advanced methodologies related to BBB imaging techniques and their applications to BBB related diseases. The current researchers have enthusiastically been developing visualization systems of BBB dynamics using advanced magnetic resonance imaging and molecular imaging techniques: (1) Novel methods that facilitate the progress of BBB imaging techniques; (2) High-resolution and high-field *ex vivo* or *in vivo* BBB imaging to better characterize properties of diseased tissues; (3) Potential applications of BBB imaging techniques in Alzheimer's disease and related dementias; (4) Analysis of BBB functions using advanced imaging techniques in the whole brain or specific brain regions between patients and healthy controls to find biomarkers for neurodegenerative diseases or try to explain their pathogenesis; and (5) Applications of BBB imaging in studying pathological processes of brain diseases that contribute to disease classification and early diagnosis.

In a comprehensive and well-written review, by Moyaert et al., they overviewed the emerging field of BBB imaging in humans by answering three key questions: (1. Disease) In which diseases could BBB imaging be useful? (2. Device) What are currently available imaging modalities for evaluating BBB function and integrity? And (3. Distribution) what is the potential of BBB imaging in different environments, particularly in resource limited settings? They highlighted the need for further advancements in imaging techniques, including validation, standardization, and the development of cost-effective methods. By addressing these challenges, BBB imaging has the potential to become a valuable clinical tool in both resource-limited and well-resourced settings.

Another comprehensive review article by Uchida et al. summarized the recent developments in BBB imaging using advanced MRI techniques in the context of Alzheimer's disease and related dementias. They described the histories and principles of non-contrast agent-based and contrast agent-based BBB imaging methodologies with a detailed comparison between them. In addition, they addressed the challenges of BBB imaging techniques and suggested future directions to develop clinically useful imaging biomarkers for Alzheimer's disease and related dementias.

Neuroinflammation has been known to play a significant role in the pathogenesis of these conditions, and BBB dysfunction is closely linked to the inflammatory processes in the brain. Lee and Funk reviewed the structural and functional changes in the BBB that occurred during the Alzheimer's pathogenesis in terms of neuroinflammation. They emphasized the importance in discerning the trajectory of BBB breakdown, aided by improving BBB imaging technologies. These advancements can help identify specific microstructural changes within the neurovascular unit and shed light on the mechanisms underlying BBB breakdown in Alzheimer's disease and related dementias.

The study conducted by Zhukov et al. investigated the BBB function and neurovascular coupling in a specific model of amyloidopathy called the 5xFAD mouse model. Using *in vivo* two-photon microscopy in the superficial cortical layers and *ex vivo* imaging across brain regions, the authors examined the BBB function and neurovascular coupling at the level of individual brain vessels in adult female 5xFAD mice leading to a better understanding of Alzheimer's pathophysiology and the development of novel therapeutic strategies.

Using both male and female 5xFAD mice, Jullienne et al. underwent *in vivo* positron emission tomography (PET) imaging with ^18^F-Fluorodeoxyglucose to assess regional glucose metabolism. They highlighted a potential mismatch between metabolic demand and vascular delivery of nutrients in the 5xFAD mouse model. This mismatch between metabolic demand and vascular supply may contribute to the progressive cognitive deficits observed in the 5xFAD mouse model. Understanding the complex interplay between vascular dysfunction and metabolic alterations might be crucial for unraveling the mechanisms underlying Alzheimer's disease and developing effective therapeutic strategies.

Overall, the collective findings presented in these manuscripts have advanced our knowledge of the BBB physiology and its implications in Alzheimer's disease and related dementias. They provide a foundation for future research and open new avenues for the development of interventions and imaging biomarkers in the field of neurodegenerative diseases.

## Author contributions

YU wrote the editorial. All authors read and approved the final editorial.

